# Influences of Splittability and Character Type on Processing of Chinese Two-Character Verb–Object Constructions

**DOI:** 10.3390/bs15111460

**Published:** 2025-10-27

**Authors:** Xiaoxin Chen, Degao Li, Wenling Ma, Meixue Zhang, Jin Wang

**Affiliations:** 1 College of Chinese Language and Literature, Qufu Normal University, Qufu 273165, China; chenxiaoxin@upc.edu.cn (X.C.); 2023230081@qfnu.edu.cn (J.W.); 2 School of Foreign Studies, China University of Petroleum (East China), Qingdao 266580, China; zhangmx@upc.edu.cn; 3 School of Foreign Languages, Liaocheng University, Liaocheng 252000, China; mawenling@lcu.edu.cn

**Keywords:** influence, splittability, character type, Chinese two-character words, verb–object constructions

## Abstract

It is theoretically accepted that Chinese two-character words (2C-words) are processed both holistically and according to their constituent characters. Given the evidence on readers’ sensitivities to the syntactic relationships between the constituent characters, however, this general view might not fully explain the 2C-word processing mechanism. As an important category of 2C-words, verb–object constructions (VOCs) exhibit significant heterogeneity in splittability, the degree of syntactic phrasalization through the insertion of other characters between the constituent characters. To examine skilled readers’ VOC processing under the influences of splittability and whether the constituent characters are bound or free characters (character type), two experiments were conducted on a cohort of college students, who were Chinese native speakers, using the lexical decision task in a repetition priming paradigm. The prime stimuli (primer type) comprised three conditions: (a) the targets themselves, (b) the targets’ transposed non-words, and (c) non-linguistic baseline symbols ‘※※’. The primers’ two constituents were presented simultaneously and sequentially in Experiments 1 and 2, respectively. A significant interaction was revealed across both experiments between splittability and character type in the participants’ performance. The main effect was significant for primer type in the participants’ performance in Experiment 1; in Experiment 2, however, the interaction was significant both between primer type and splittability in the participants’ performance and between primer type and character type in their reaction times. In addition to confirming the general view, skilled readers might inevitably experience syntactic and semantic combinations of the constituent characters in their processing of VOCs.

## 1. Introduction

In Chinese, the fundamental orthographic units are Chinese characters. Almost all characters are meaningful on their own, and the commonly used ones frequently join with one another to formulate two-character words (2C-words) or multi-character words. By words, we mean the lexical entries in a standard Chinese dictionary. Notably, 72% of the commonly used words (State Language Commission, 2008) are 2C-words, which are theoretically regarded as being processed both holistically and according to the constituent characters in cognitive tasks (e.g., Taft & Zhu, 1997; Peng et al., 1999b; X. Zhou & Marslen-Wilson, 2000). Given the syntactic relationships between the constituent characters (e.g., J. Zhou, 2004), more studies on the mechanism of 2C-word processing are needed. The present study attempts to address this issue through two experiments adopting the lexical decision task (LDT) in a repetition priming paradigm.

### 1.1. 2C-Word Processing

Around the end of the last century, prominent theories had been proposed concerning the co-existing representations for 2C-words and their constituent characters. According to the Multilevel Interactive Activation Model (Taft & Zhu, 1997), for example, readers have representations that are hierarchically organized for 2C-words and their constituent characters. Activation of representations at the character level is transmitted to the word level, and vice versa. The Inter- and Intra-Connection Model (Peng et al., 1999b) argues for facilitating links between the word- and character-level representations for semantically transparent words but for inhibitory links between the two levels of representations for semantically opaque words. The Lexical Representation Framework for Chinese Compound Word (X. Zhou & Marslen-Wilson, 2000) also assumes inter-dependence between semantic representations for 2C-words and their constituent characters in 2C-word processing. In alignment with these theoretical points, numerous studies have been conducted into the influences of word- (e.g., P. Chen et al., 2015; B. G. Chen & Peng, 1998; Fan et al., 2017; D. Li et al., 2020; Shen & Li, 2016; C. M. Wang & Peng, 2000; J. Wang et al., 2024; X. Zhou & Marslen-Wilson, 1994) and character-level (e.g., Huang & Lee, 2018; D. Li et al., 2020; Liang et al., 2016; H. X. Meng et al., 2014; Peng & Wang, 1997; Taft, 1994) features on 2C-word processing. These present the general view that 2C-words are processed both as whole entities and according to their constituent characters.

However, the general view does not seem to fully explain 2C-word processing, given that most 2C-words are characterized as syntactic compositions of their constituent characters. According to J. Zhou (2004), for example, 96.6% of the 32,000 2C-word items (4th Edition, 2002 Enlarged and Revised Edition) can be categorized as structures of coordination (SoCs), structures of modification (SoMs), verb–object constructions (VOCs), structures of predicates (SoPs), or supplementary structures (SSs). In accordance with this linguistic observation, evidence is available on readers’ sensitivities to the syntactic relationships between the constituent characters (SRCs) in 2C-word processing.

For example, B. Zhang and Peng (1992) conducted an LDT on a cohort of skilled readers with a sample of SoCs and SoMs as the targets. The participants’ responses were enhanced by the increase in the frequency of both the first and the second characters for the SoCs. For the SoMs, however, their performance was facilitated by the increase in the frequency of the head character but not the modifier character. Gao et al. (2022) adopted the technique of event-related potentials to record participants’ responses in the LDT, with a sample of SoCs, SoMs, VOCs, SoPs, SSs, and their pseudo forms as the targets. With one of its constituent characters (e.g., ‘笑’/xiao4, *laugh*) replaced with another character (e.g., ‘哭’/ku1, *cry*), a 2C-word (e.g., ‘发笑’/fa1xiao4, *making one laugh*) became a pseudo 2C-word (e.g., ‘发哭’/fa1ku1), which replicated the SRC of the word. The P600 component had the largest amplitude for the non-word targets, the second largest amplitude for the pseudo words, and the smallest amplitude for the word targets. P600 indicates processing difficulties in syntactic processing (J. X. Gu & Yang, 2010). Indeed, even child students (Xiao & Liang, 2017) and adult learners with Chinese as a second language (Y. H. Xu, 2014) can develop a kind of SRC awareness after a few years of Chinese literacy study. Given the empirical demonstrations of readers’ sensitivities to SRCs, a targeted investigation on 2C-words that belong to the specific category of SoMs, SoCs, VOCs, SoPs, or SSs is warranted.

Moreover, students are required to prioritize mastering a few thousand of the most frequently used characters, according to the Compulsory Education Chinese Language Curriculum Standards (China Ministry of Education, 2022). In their first years of literacy learning, they continuously practice via different exercises, becoming familiar with the fact that a commonly used character joins with many others to form 2C-words. Even a third-year student can be aware that a 2C-word is a combination of two individual characters and actively use the structural rules of words and characters to infer the meanings of new words (Xia et al., 2022). Moreover, a word item is often explained through its constituent characters in a standard Chinese dictionary, and Chinese readers usually comprehend a newly encountered 2C-word by combining his or her understanding of the constituent characters.

Learning experiences determine one’s memory (Andrews et al., 2009) and may affect one’s cognitive activities. For example, toddlers are usually taught to associate category names (e.g., fruits) with exemplars of middle (e.g., pears and oranges) rather than high typicality (e.g., apple and banana) (Shipley et al., 1983). Likely because of such experiences of category name learning at an early age, college students’ response latencies were significantly shorter in a semantic categorization task on category names in written Chinese primed by exemplar pictures of middle rather than high typicality (J. Xu et al., 2015). Similarly, deaf and hard-of-hearing (DHH) students learn category names in written language through associations with the corresponding exemplars of middle typicality. Likely due to this reason, DHH adolescents had higher accuracies in a semantic categorization task on category names primed by exemplar words of middle rather than high typicality in written Korean (D. Li et al., 2011). In other words, as a result of their experiences in literacy learning, skilled native Chinese readers may have developed a mechanism for 2C-word processing that involves SRC awareness.

### 1.2. Research Question

Due to historical reasons, many 2C-words are splittable, originating through the path of lexicalization (e.g., Dong, 2002) or through a process of de-lexicalization (e.g., Z. J. Li, 2006). Among the five categories of 2C-words, VOCs are particular in that most of them turn into phrases with other characters inserted between the constituent characters (Zhu, 1982). In fact, VOCs vary in the extent to which other characters can be inserted between the constituent characters (e.g., H. J. Wang, 2000). For example, the VOC ‘帮忙’/bang1mang2 (*help*) becomes a meaningful phrase with ‘了’/le, ‘着’/zhe, ‘过’/guo4, ‘个’/ge4, ‘些’/xie1, ‘倒’/dao4, ‘上’/shang4 or ‘下’/xia4 inserted between its constituent characters ‘帮’/bang1 and ‘忙’/mang2. However, the possibility is much smaller for the VOC ‘走私’/zou3si1 (*smuggle*) to become a meaningful phrase with other characters inserted between its constituent characters ‘走’/zou3 and ‘私’/si1.

The characters that can be inserted between the constituents belong to the category of complementary elements, modifying/restrictive elements, both complementary and modifying/restrictive elements, verb reduplication, or object fronting (K. Meng & Wang, 2017). Thus, there are differences among VOCs in the degree to which other characters can be inserted between the constituent characters (splittability). Those with a high level of splittability are recognized as splittable words (e.g., Dong, 2002). Since the way a VOC can be split is often determined by the linguistic intuition or conventions of the language community (Xie, 2001), one may determine splittability scores through subjective evaluation.

Obviously, there are more possibilities for the constituent characters of a high-splittability rather than a low-splittability VOC to be semantically or syntactically associated with each other. There are potentially stronger semantic or syntactic associations between the constituent characters of a high- rather than low-splittability VOC. That is, high-splittability VOCs are more likely than low-splittability ones to be seen as compositions of their constituent characters, and readers’ VOC processing would probably be affected by the word-level feature of splittability.

Meanwhile, commonly used characters fall into the category of free or bound characters (Zhu, 1982). Free characters are those that can be used as one-character words in certain contexts; bound characters are those that cannot be used as words on their own right in any context. Indeed, 77.4% and 22.6% of the 2500 most commonly used characters (State Language Commission, Chinese Character Division, 1988) are free and bound characters, respectively (C. Wang, 2017). In addition to their larger proportion of the total number of characters, free characters are more likely to develop individual representations than bound characters (S. S. Zhang & Yang, 2012); 2C-words with free characters as the constituent characters are more transparent in semantics than those with bound characters as the constituent characters (J. X. Li, 2011). Therefore, 2C-word processing might be influenced by the character-level feature of whether the constituent characters are free or bound characters.

In fact, the constituent characters’ character type plays a role in mediating VOCs’ splittability (M. Y. Li, 2014). This is because 2C-words with free characters as the constituent characters are more loosely structured than those with bound characters as the constituent characters (Xie, 2001). Meanwhile, there are splittable words whose constituent characters belong to the category of bound characters. For example, the first and second characters of ‘造孽’/zao4nie4 (*commit a sin*) are free and bound characters, respectively; the constituent characters of ‘毕业’/bi4ye4 (*graduate*) are both bound characters. Therefore, splittability and character type may interact with each other in their influences on VOC processing.

The above-mentioned speculations acquire heightened theoretical significance when the prominent feature of 2C-words is taken into consideration: that the constituent characters have fixed relative positions. In total, 96.7% of the commonly used 2C-words turn into non-words upon transpositions of their constituent characters (J. Gu et al., 2015). Indeed, studies are available on readers’ sensitivity to the constituent characters’ relative positions in 2C-word processing. For example, J. Q. Zhang (2009) conducted an LDT in a repetition paradigm at the stimulus onset asynchrony (SOA) of 100 ms. A priming effect was revealed when the primers were the 2C-word targets themselves or were the targets’ transposed non-words. Importantly, the priming effect was significantly larger for the word than for the non-word primers. E. J. Xu and Sui (2018) achieved a similar result at the SOA of 80 ms using a similar paradigm to that in J. Q. Zhang (2009); Bian et al. (2010) and Liu et al. (2019) also indicated skilled readers’ processing difficulty on the encounter of transposed non-words in sentence reading.

One reason why transposed non-words are more difficult to process than their corresponding words might originate from the violation of proper SRCs. For example, when the constituent characters of the VOC ‘洗澡’/xi3zao3 (*to take a bath*) are transposed, the resulting two-character cluster ‘澡洗’ violates logical coherence if read from left to right. Readers will find it more difficult to process the non-word ‘澡洗’ than the word ‘洗澡’ due to the significant processing costs. Consequently, the research question of the present study is as follows: How do splittability and character type interact with the transposition of constituent characters in VOC processing?

### 1.3. Present Study

Two experiments were conducted on a cohort of college students who were Chinese native speakers, using the LDT in a repetition priming paradigm, in which the VOC targets would differ in splittability and character type, and the primers would be the targets themselves or the targets’ transposed non-words (primer type). Given the likelihood of readers’ SRC sensitivities in 2C-word processing, interactions would be revealed between the influences of splittability, character type, and primer type on participants’ performance. The anticipated results were positioned to provide new evidence of skilled readers’ SRC sensitivities in 2C-word processing, taking into consideration the influences of splittability and character type.

## 2. Experiment 1

In Peng et al. (1999a), a cohort of skilled readers was required to make lexical decisions on the targets (e.g., ‘西装’/xi1zhuang1, *suit*) in a priming paradigm. The targets were preceded by words (e.g., ‘领带’/ling3dai4, *tie*) that were semantically associated with the targets, or the transposed forms (e.g., ‘带领’/dai4ling3, *to lead*) of the word primers. There are approximately 800 commonly used 2C-words that become other words upon transpositions of the constituent characters (Xing, 2006). At the SOA of 57 ms, the researchers observed a significantly larger priming effect for the word primers than for the corresponding transposed forms. That is, skilled readers’ awareness of the constituent characters’ relative positions in their semantic processing of a 2C-word can be detected in a duration of around 60 ms. Thus, the SOA was determined to be 60 ms in the present study.

### 2.1. Method

#### 2.1.1. Participants

We recruited 108 students who were Chinese native speakers (70 females, 38 males; mean age = 18.93 years, age range = 18~21 years) as the participants via a flyer advertisement on the campus of Qufu Normal University. The participants were from different academic backgrounds and had normal or corrected-to-normal vision. They signed a written consent form in accordance with the Declaration of Helsinki and received RMB 20 (USD 2.78) as a reward for participation. The study was approved by the Ethics Committee of Qufu Normal University.

#### 2.1.2. Materials

We used the data from https://osf.io/dx6uq/?view_only=960848718e634f96b6c52b2085d20464 (accessed on 15 January 2024) as the pool to select 86 VOCs (see Appendix A), and the SRC types were determined with reference to Feng (2018). The character types of the constituent characters were determined with reference to C. Q. Li (2008) and the *Modern Chinese Dictionary* (the Seventh Edition). The first and second constituent characters of the VOCs are called the verb and object characters, respectively. All the verb characters belonged to the category of free characters, but 40 and 46 of the object characters belonged to the categories of bound and free characters, respectively. The splittability score of each VOC was evaluated on a 5-point Likert scale (1 = *very difficult to insert other characters between the constituent characters of an item to make a meaningful phrase*; 5 = *very easy to insert other characters between the constituent characters of an item to make a meaningful phrase*) by another group of 26 students who were Chinese native speakers. Similar to the participants of the experimental task, the raters were from different academic backgrounds and were randomly recruited from China University of Petroleum (East China). There were 20 and 23 high-splittability VOCs, the object characters of which were bound and free characters, respectively, and 20 and 23 low-splittability VOCs, the object characters of which were bound and free characters, respectively. As displayed in Table 1, the splittability scores of the low-splittability VOCs were significantly lower than those of the high-splittability VOCs. Pairwise t-tests suggested that there were no significant differences among the four groups of VOCs in 14 word- and 13 character-level features.

Each group of VOCs was divided into three largely equal sub-groups. Adopting the Latin square design, three sets of critical prime-target stimuli were created: every VOC appeared once in each set as the target, paired with itself as the primer in set one, with its transposed non-word as the primer in set two, and with the meaningless symbol ‘※※’ as the primer in set three. ‘※※’ was used as the baseline primer, similar to previous studies on word processing in alphabetic languages, in which a string of ‘X’ was adopted (e.g., Neely et al., 1989). Forty-three 2C-words, which did not belong to the category of VOCs and were treated as yes-response targets, were divided into three largely equal groups. Groups one, two, and three were paired with the targets themselves, their transposed non-words, and the meaningless symbol, respectively, as the primers. The 43 yes-response primer-target filler stimuli were randomly mixed with each of the three sets of critical materials to create three sets of yes-response stimuli. One hundred twenty-nine two-character non-words were created as the no-response targets. The resulting first, second, and third sets of the no-response targets were paired with the targets themselves, the transposed forms of the targets, and the meaningless symbol, respectively, as the primers. The 129 no-response primer-target stimuli were randomly mixed with each of the three sets of yes-response stimuli to create three blocks of materials.

#### 2.1.3. Design

The design formed a 2 (splittability: the targets were VOCs of a high or low level of splittability) × 2 (character type: the object characters were free or bound characters) × 3 (primer type: the primers were the meaningless symbol ‘※※’, the targets themselves, or the transposed non-words of the targets) factorial of repeated measurements. The dependent variables were participants’ response errors and reaction latencies for the correctly responded trials (RTs). The design required a minimum of 54 participants for an effect size of 0.25, at a significance level of 0.05 with a power of 0.95, and there were 108 participants in this experiment.

#### 2.1.4. Procedure

The measurements were collectively implemented in the Laboratory of Psycholinguistics of the College of Chinese Language and Literature, Qufu Normal University, with E-Prime 3.0 to present stimuli and record participants’ responses. Each participant was randomly assigned to run the task on material block one, two, or three. They were each seated in front of a computer with their eyes 60 cm away from the computer screens. In each trial, a red fixation cross (‘+’) was shown in the center of the screen for 700 ms. Next, a two-character cluster was shown at the screen center for 40 ms. After the mask ‘▓▓’ was shown for 20 ms, the target appeared for 2000 ms or until a key-press response was received at the place where the primer disappeared. Participants were required to decide whether or not the target was a meaningful word by pressing one of the two designated keys on the keyboard. The next trial began after the screen was blank for 1000 ms. The experiment lasted 14 min.

### 2.2. Results

The data were excluded from the trials if the reaction times were shorter than 200 ms or longer than the overall mean plus 3 *SD*s. In total, 2.00% of the collected data were deleted, and the error rate and the reaction time (RT) scores for the correct responses are summarized in Table 2.

A mixed-effects binary logistic regression analysis of the error scores (1 = incorrect response, 0 = correct response) and a mixed-effects regression analysis of the logged RTs were conducted using lme4 (Bates et al., 2015) in R (R Development Core Team, 2012). Only significant effect results are reported here. Original data and analysis code are available at https://osf.io/qfuwa/?view_only=e800c515dd084e539c62fc1b0ee998d8 (accessed on 28 June 2025).

#### 2.2.1. Error Scores

The main effect was significant for primer type. The participants had a significantly lower probability of making errors (PrbE) when the primers were the targets themselves (*M* = 0.031, 95% CI = 0.021~0.045) than when they were transposed non-words (*M* = 0.045, 95% CI = 0.032~0.065) (*β* = 0.390, *SE* = 0.112, *z* = 3.479, *p* = 0.0015) or the meaningless symbol (*M* = 0.047, 95% CI = 0.032~0.067) (*β* = 0.420, *SE* = 0.112, *z* = 3.754, *p* = 0.0005). Probability scores (ranging from 0 to 1) were obtained through reverse transformations of the logit scores. The interaction was significant between splittability and character type. Simple analyses were conducted using the emmeans package (Lenth et al., 2025). The participants’ PrbE was significantly lower in the condition of free (*M* = 0.022, 95% CI = 0.015~0.033) than bound (*M* = 0.053, 95% CI = 0.037~0.076) characters for the high-splittability targets (*β* = 0.901, *SE* = 0.141, *z* = 6.414, *p* < 0.0001). It was significantly lower for the high-splittability than for the low-splittability (*M* = 0.051, 95% CI = 0.035~0.073) targets in the condition of free characters (*β* = 0.863, *SE* = 0.137, *z* = 6.298, *p* < 0.0001).

#### 2.2.2. RT Scores

The main effect was significant for primer type. The participants had significantly shorter RTs when the primers were the targets themselves (*M* = 620 ms, 95% CI = 602~639 ms) than when they were transposed non-words (*M* = 672 ms, 95% CI = 652~692 ms) (*β* = 0.080, *SE* = 0.006, *t* = 13.543, *p* < 0.0001) or the meaningless symbol (*M* = 706 ms, 95% CI = 686~728 ms) (*β* = 0.130, *SE* = 0.006, *t* = 22.078, *p* < 0.0001). Their RTs were significantly shorter when the primers were transposed non-words than when they were the meaningless symbol (*β* = 0.050, *SE* = 0.006, *t* = 8.519, *p* < 0.0001). The interaction was significant between splittability and character type. The participants’ RTs were significantly shorter in the condition of free (*M* = 650 ms, 95% CI = 631~669 ms) than bound (*M* = 677 ms, 95% CI = 657~698 ms) characters for the high-splittability targets (*β* = 0.041, *SE* = 0.007, *t* = 5.926, *p* < 0.0001). They were significantly shorter for the high-splittability than for the low-splittability (*M* = 664 ms, 95% CI = 644~684 ms) targets in the condition of free characters (*β* = 0.022, *SE* = 0.007, *t* = 3.297, *p* = 0.0010).

### 2.3. Discussion

Experiment 1 yielded two results. First, the participants performed significantly better when the primers were words than when they were transposed non-words, suggesting that they considered the primers as whole entities. Their representations for the targets were pre-activated more deeply when the primers were the targets themselves rather than when they were the corresponding transposed non-words. Moreover, their RTs were significantly shorter with the non-word than with the symbol primers. In comparison to the symbol primers, their processing of the constituent characters of the non-word primers must have resulted in pre-activation of the representations for the targets to a certain extent. In sum, the main effect of primer type on the participants’ performance simply confirmed the general view.

Different from expectations, however, no significant interaction was observed between primer type and the other two variables. The participants’ SRC sensitivities with the primers, whose constituent characters were simultaneously presented for a duration of 60 ms, might not have been strong enough to affect their responses.

Second, the participants’ performance was interactively affected by splittability and character type, which mainly suggested their processing of the targets. On the one hand, the participants performed significantly better on the targets with a high level of splittability (compared to those with a low level) when the second characters belonged to the category of free characters. When the second characters belonged to the category of bound characters, however, their responses were not significantly different between the targets of high and low levels of splittability. On the other hand, they performed significantly better when the second characters were free characters for the high-splittability targets. Considering the targets with a low level of splittability, however, they were not sensitive to the changes in the character type of the second character.

In particular, this result may imply more than simply confirming the general view. Given that there are splittable words that consist of bound characters, the participants should have been aware of the targets’ differences in splittability in the bound-character condition, which was not the case. This initiated the necessity of Experiment 2.

In Lv et al.’s (2022) first experiment, in which the constituent characters of the 2C-word targets were simultaneously presented, the participants’ responses in lexical decisions were not affected by changes in the visual complexity of the first characters. In their second experiment, the second characters of the targets were presented after the first characters; the participants’ reaction latencies tended to be significantly affected by the visual complexity of the first characters. In comparison to Experiment 1 in Lv et al.’s (2022), in which the target and its constituent characters might be processed in parallel, the participants were more able to focus on the first character in Experiment 2.

Similar to how the targets were presented in Experiment 2 in Lv et al. (2022), in the present study, the constituent characters of the primer were shown one after another in Experiment 2, using the same task as Experiment 1. Participants were forced to process the first character more deeply than the second one, and interactions would probably be revealed between the primer type and the other two variables in the measurements.

## 3. Experiment 2

### 3.1. Method

The same task as Experiment 1 was adopted, except that the constituent characters of the primers were presented sequentially. The purpose was to reveal whether participants’ sequential processing of the primers’ constituent characters would be affected by splittability and/or character type. One hundred and eight participants were recruited in the same way as in Experiment 1. The materials, design, and procedure were the same as Experiment 1, except the first and second characters of the primer were each presented for 20 ms sequentially in a trial.

### 3.2. Results

The data were trimmed in the same way as in Experiment 1, and the error-rate and RT scores are summarized in Table 2. The data were analyzed in the same way as in Experiment 1.

#### 3.2.1. Error Scores

The interaction was significant between splittability and character type. The participants had a significantly lower PrbE in the condition of free (*M* = 0.037, 95% CI = 0.026~0.053) than bound (*M* = 0.075, 95% CI = 0.054~0.104) characters for the high-splittability targets (*β* = 0.752, *SE* = 0.121, *z* = 6.230, *p* < 0.0001). Their PrbE was significantly lower for the high-splittability than for the low-splittability targets (*M* = 0.072, 95% CI = 0.052~0.099) in the condition of free characters (*β* = 0.698, *SE* = 0.118, *z* = 5.928, *p* < 0.0001) but was significantly higher for the high-splittability than for the low-splittability targets (*M* = 0.059, 95% CI = 0.042~0.083) in the condition of bound characters (*β* = 0.261, *SE* = 0.111, *z* = 2.347, *p* = 0.0189). The interaction was significant between splittability and primer type. The participants’ PrbE was significantly higher when the primers were the meaningless symbol (*M* = 0.083, 95% CI = 0.059~0.115) than when they were the targets themselves (*M* = 0.054, 95% CI = 0.038~0.077) for the low-splittability targets (*β* = 0.451, *SE* = 0.132, *z* = 3.419, *p* = 0.0018).

#### 3.2.2. RT Scores

The interaction was significant between splittability and character type. The participants’ RTs were significantly shorter in the condition of free (*M* = 646 ms, 95% CI = 626~666 ms) than bound (*M* = 663 ms, 95% CI = 643~685 ms) characters for the high-splittability targets (*β* = 0.027, *SE* = 0.007, *t* = 3.965, *p* = 0.0001). Their RTs were significantly shorter for the high-splittability than for the low-splittability targets (*M* = 662 ms, 95% CI = 642~683 ms) in the condition of free characters (*β* = 0.024, *SE* = 0.006, *t* = 3.805, *p* = 0.0001).

The interaction was significant between splittability and primer type. The participants’ RTs were significantly shorter for the high-splittability (*M* = 647 ms 95% CI = 626~667 ms) than for the low-splittability (*M* = 663 ms, 95% CI = 642~684 ms) targets when the primers were transposed non-words (*β* = 0.024, *SE* = 0.008, *t* = 2.949, *p* = 0.0032). Their RTs were significantly longer when the primers were the meaningless symbol (*M* = 683 ms, 95% CI = 662~705 ms) than when they were transposed non-words (*β* = 0.056, *SE* = 0.008, *t* = 6.884, *p* < 0.0001) or the targets themselves (*M* = 634 ms, 95% CI = 614~655 ms) (*β* = 0.074, *SE* = 0.008, *t* = 9.147, *p* < 0.0001) for the high-splittability targets. Similarly, for the low-splittability targets, their RTs were significantly longer when the primers were the meaningless symbol (*M* = 696 ms, 95% CI = 675~719 ms) than were transposed non-words (*β* = 0.050, *SE* = 0.008, *t* = 6.141, *p* < 0.0001) or the targets themselves (*M* = 632 ms, 95% CI = 612~653 ms) (*β* = 0.097, *SE* = 0.008, *t* = 11.859, *p* < 0.0001). Their RTs were significantly longer when the primers were transposed non-words than were the targets themselves (*β* = 0.046, *SE* = 0.008, *t* = 5.728, *p* < 0.0001).

The interaction was significant between character type and primer type. The participants’ RTs were significantly shorter in the condition of free (*M* = 626 ms, 95% CI = 606~646 ms) than bound (*M* = 642 ms, 95% CI = 622~663 ms) characters when the primers were the targets themselves (*β* = 0.025, *SE* = 0.008, *t* = 3.088, *p* = 0.0020). In the condition of free characters, the participants’ RTs were significantly longer when the primers were the meaningless symbol (*M* = 683 ms, 95% CI = 662~705 ms) than were transposed non-words (*M* = 654 ms, 95% CI = 634~675 ms) (*β* = 0.043, *SE* = 0.008, *t* = 5.520, *p* < 0.0001) or the targets themselves (*β* = 0.088, *SE* = 0.008, *t* = 11.305, *p* < 0.0001) and were significantly longer when the primers were transposed non-words than were the targets themselves (*β* = 0.045, *SE* = 0.008, *t* = 5.776, *p* < 0.0001). In the condition of bound characters, their RTs were significantly longer when the primers were the meaningless symbol (*M* = 697 ms, 95% CI = 675~720 ms) than when they were transposed non-words (*M* = 655 ms, 95% CI = 634~676 ms) (*β* = 0.063, *SE* = 0.008, *t* = 7.418, *p* < 0.0001) or the targets themselves (*β* = 0.083, *SE* = 0.008, *t* = 9.757, *p* < 0.0001).

### 3.3. Discussion

The same pattern of interaction as Experiment 1 was observed between splittability and character type in the participants’ performance. In addition to this sound result, Experiment 2 did achieve a significant interaction between primer type and splittability and an interaction between primer type and character type in the RT scores. In addition to confirming the general view, these two interactions revealed two notable points: The RTs were significantly shorter when the primers were the targets themselves rather than the targets’ transposed non-words, for the low- but not high-splittability VOCs, and in the condition of free but not bound characters. Aligning with expectations, these results must have been due to the participants’ increased attentional focus on the first presented characters.

To understand these results, we would take the following as presuppositions, given the existence of varieties among 2C-words in being taken as syntactic compositions (J. Zhou, 2004) and semantic combinations (Fu, 1985) of their constituent characters. In comparison with the high-splittability VOCs, the low-splittability ones might have been more likely to be taken as orthographic wholes by the participants; in comparison with the VOCs in the condition of free characters, those in the condition of bound characters might have been more likely to be taken as semantic wholes.

#### 3.3.1. Splittability and Primer Type

The participants’ RTs were significantly longer for the low-splittability targets preceded by the non-word than word primers. The non-word primers were less likely than the word primers to have triggered pre-activation of the representations for the targets. This was because the participants might have taken the low-splittability VOCs as orthographic wholes, and there was a mismatch between the non-word primers and the targets in orthography.

At the high level of splittability, however, the participants’ RTs were not significantly different between the word and non-word primers. The relative positions of the constituent characters did not affect their pre-activation of the representations for the targets. Their processing of the non-word primers seemed to lead to the same amount of priming effect as that of the word primers. This result might not have been due to the higher degree of morphological transparency and/or greater familiarity with constituent characters for the high- rather than low-splittability VOCs. Otherwise, a similar pattern of results would have been revealed in Experiment 1, which was not the case. Alternatively, the participants must have processed the first presented characters more deeply than the second presented ones. Likely due to this difference in depth of processing between the constituent characters, the participants’ SRC sensitivities played a role in triggering pre-activation of representations for the high-splittability VOCs.

#### 3.3.2. Primer Type and Character Type

When the object characters of the targets were bound characters, the participants’ RTs were not significantly different between the word and transposed non-word primers. Their processing of the object characters (in the condition of non-word primers) was as capable as their processing of the verb characters (in the condition of word primers) in activating representations for the corresponding words. This is analogous to the case that perceiving either the head or the tail of a horse results in representation activation for the specific type of animal. As presupposed, the VOCs with bound characters as the object characters might be taken as fixed entities in semantics.

When the object characters were free characters, however, the participants’ RTs were significantly longer for the transposed non-word than for the word primers. This result could not be explained by the differences between free and bound characters per se. Otherwise, the interaction should have been significant between character type and primer type in Experiment 1, which was not the case. One explanation might be as follows. In the condition of word primers, their processing of the verb characters contributed to activating the representations for the 2C-words, largely because of their SRC sensitivities. In the condition of non-word primers, however, their processing of the object characters might not have contributed to activating the representations for the corresponding 2C-words, largely due to the lack of SRC sensitivities.

#### 3.3.3. Integrated Reflections

The speculations on the participants’ SRC sensitivities with the primers in Experiment 2 seem to be well confirmed by the sound interaction between splittability and character type in the participants’ performance across the two experiments. Because of the enhancement of their SRC sensitivities, the participants might have experienced rich syntactic and semantic combinations of the constituent characters and performed the best on the high-splittability targets in the condition of free characters; because of the least enhancement of their SRC sensitivities, however, they might have experienced little syntactic and semantic combinations of the constituent characters and performed the most poorly on the low-splittability targets in the condition of bound characters. The absence of interactions between primer type and the other two variables in Experiment 1 might indicate that skilled readers’ SRC sensitivities to a VOC, when its constituent characters are presented simultaneously, may not begin to function in the first several tens of milliseconds of its presentation.

## 4. General Discussion

To examine the influences of splittability and character type on VOC processing, two experiments were conducted on a cohort of skilled readers via an LDT in a repetition priming paradigm. The VOC targets differed in splittability and character type, with the targets themselves, the targets’ transposed non-words, or the meaningless symbols as the primers. The two characters of the primers were presented simultaneously and sequentially in Experiments 1 and 2, respectively. A significant interaction was consistently revealed in the two experiments between splittability and character type in the participants’ performance. The main effect of primer type was significant in the participants’ performance in Experiment 1. In Experiment 2, however, the interaction was significant between primer type and splittability in the participants’ performance and between primer type and character type in their RTs.

Aligning with expectations, these results seem to replicate the inevitability of skilled readers’ SRC sensitivities in 2C-word processing (e.g., Gao et al., 2022). Thus, further research is needed to determine how the role of SRC sensitivities might be connected to the general view. Nevertheless, the present study strongly suggests that high-splittability VOCs with free characters as the second characters might be processed as syntactic and semantic combinations of the constituent characters. Since the number of free characters is more than three times that of bound characters in the *General and Standard Chinese Characters Table* (Shao, 2022), for example, and most VOCs are splittable (e.g., B. Zhang, 2005), a VOC has a large probability of being processed as a syntactic and semantic combination of its constituent characters.

Therefore, in future studies, more evidence needs to be obtained on the constituent–character combination in 2C-word processing, in which the semantic relationships between the constituent characters of a specific category of 2C-words are manipulated. For example, in some high-splittability VOCs composed of free characters (e.g., ‘念书’/nian4shu1, *study*), the second character serves as the real object of the first character (rVOCs). For other high-splittability VOCs that are composed of free characters, however, the second characters are pseudo-objects of the first characters (e.g., ‘养病’/yang3bing4, *recuperate*) (pVOCs). If readers inevitably combine the constituent characters in VOC processing, as indicated in the present study, then their responses will be affected by whether stimuli are rVOCs or pVOCs in a cognitive task.

The word-level feature of splittability might not apply to SoCs, SoMs, SSs, SoPs, or VOCs, but character type and semantic relationships between the constituent characters can be manipulated. Specific patterns emerge for different categories of 2C-words, depending on when syntactic and semantic combinations of the constituent characters occur during readers’ recognition of VOCs, SoCs, SoMs, SSs, and SoPs.

Practically, the word-level feature of splittability and the character-level feature of whether the constituents are free or bound characters may be taken into consideration in Chinese literacy education. For example, guiding students to perform exercises on how a VOC is splitable and how character type functions in character combination in semantics may help shape their word learning strategies and strengthen their interest in vocabulary development.

The present study has several limitations. First, the participants were very familiar with the stimuli, which should have induced a ceiling effect. Future studies employing VOCs whose constituent characters are beyond the 2500 most commonly used characters may result in more insightful findings; using VOCs that are of middle frequency as the targets, for example, may yield more details on the interactive influences of splittability and character type. Second, the SOA was relatively short, and the priming effect may have potentially introduced noise in the data. Future studies may employ longer SOAs to ensure semantic and syntactic processing of the primers. Third, the baseline primer ‘※※’ might have been too distinct from Chinese characters. For example, two characters in Korean Hangul would probably be a better choice.

In conclusion, in addition to confirming the general view that 2C-words are processed both as wholes and according to the constituent characters, skilled readers might inevitably experience syntactic and semantic combinations of the constituent characters in their recognition of VOCs. However, more studies are needed as to how to connect readers’ SRC sensitivities to the general view.

## Figures and Tables

**Table 1:** Feature Scores of the Critical Materials.

		High Splittability				Low Splittability				High Splittability	Low Splittability	Free Character	Bound Character
		Free Character		Bound Character		Free Character		Bound Character		Free-Bound Character	Free-Bound Character	High/Low Splittability	High/Low Splittability
		*M*	*SD*	*M*	*SD*	*M*	*SD*	*M*	*SD*	*t-Value*	*t-Value*	*t-Value*	*t-Value*
Left Character	AoA	1.39	0.14	1.38	0.09	1.38	0.20	1.45	0.30	−0.43	0.74	0.28	−0.87
	Frequency	4.25	0.67	4.10	0.38	4.05	0.26	4.07	0.40	−0.60	0.18	0.68	−0.14
	Concreteness	5.12	0.13	5.01	1.56	4.83	0.52	4.62	0.95	−0.38	−0.60	1.63	1.57
	Imageability	3.80	0.24	3.75	0.67	3.52	0.25	3.64	1.50	−0.23	0.27	1.54	0.83
	Sensory Experience Arousal	3.10	0.30	2.81	0.86	3.05	0.35	3.03	0.65	−1.33	−0.04	0.40	−0.71
	Valence	4.12	0.47	4.20	0.13	4.21	0.05	4.33	0.82	0.53	0.60	−0.74	−0.86
	Emotional Experience Arousal	2.99	1.42	2.77	1.17	2.72	0.65	2.78	0.06	−0.60	0.12	0.98	0.41
	Number of Strokes	7.83	2.12	8.30	2.12	8.61	0.00	9.10	0.00	0.64	0.72	−0.92	−0.83
	Number of Components	1.99	0.40	1.92	0.16	2.05	0.19	1.90	0.07	−0.23	−1.14	−0.66	0.23
	Number of Meanings	5.48	0.71	6.50	2.12	6.00	0.71	5.55	0.71	1.23	−0.43	−0.73	0.93
	Number of Words (L)	26.96	4.24	41.20	19.09	23.30	4.95	30.45	3.54	1.44	1.00	0.59	0.93
	Number of Words (R)	16.09	13.44	21.95	4.95	21.65	4.24	21.25	24.75	0.91	−0.02	−1.16	−0.04
	Semantic Association Strength	3.97	0.00	3.68	0.08	3.87	0.23	3.84	0.14	−1.42	−0.13	0.65	−0.67
Right Character	AoA	1.53	0.21	1.63	0.30	1.43	0.24	1.55	0.21	0.96	1.34	1.26	0.99
	Frequency	3.59	0.21	3.54	1.07	3.53	0.81	3.63	0.49	−0.46	0.54	0.20	−0.87
	Concreteness	5.89	0.57	5.61	0.46	5.66	0.57	5.42	0.37	−1.17	−0.88	0.95	0.93
	Imageability	4.19	0.52	3.95	1.04	4.09	0.71	3.96	0.08	−0.61	−0.46	0.31	0.13
	Sensory Experience Arousal	3.53	0.47	3.15	0.48	3.48	1.05	3.25	1.07	−1.10	−1.08	−0.03	−0.03
	Valence	3.94	0.04	4.19	0.85	4.21	1.05	4.52	0.40	0.93	1.59	−0.82	−1.18
	Emotional Experience Arousal	3.19	1.24	3.20	0.98	3.18	0.39	3.33	0.59	0.48	0.31	−0.28	−0.17
	Number of Strokes	10.04	4.95	9.00	0.00	9.39	0.00	9.05	1.41	−1.14	−0.49	0.73	0.06
	Number of Components	2.12	0.95	2.23	0.41	2.08	0.49	2.21	0.03	1.24	1.05	0.03	0.40
	Number of Meanings	4.48	4.24	3.95	2.12	3.61	0.71	3.20	0.71	−0.99	−0.55	1.65	0.91
	Number of Words (L)	20.00	74.25	11.50	7.07	15.35	18.38	12.25	6.36	−1.70	−0.59	0.98	−0.31
	Number of Words (R)	28.57	57.98	26.90	33.23	20.00	4.95	19.20	3.54	−0.72	−0.11	1.58	1.10
	Semantic Association Strength	4.29	0.54	4.50	0.00	4.26	0.29	4.34	0.27	1.58	0.08	−0.13	1.34
Word	Splittability	2.78	1.25	2.72	0.68	2.05	0.63	1.95	0.84	−0.36	−1.08	8.14 ***	7.62 ***
	AoA	2.57	0.62	2.77	0.44	2.38	0.16	2.59	0.66	1.11	1.58	1.68	1.14
	Frequency	1.26	1.12	1.36	0.17	1.41	0.69	1.36	1.00	0.84	−0.21	−1.11	−0.10
	Concreteness	5.24	0.95	5.02	0.20	5.22	0.54	5.27	0.11	−0.91	0.48	0.28	−1.18
	Imageability	4.37	1.66	4.29	0.23	4.25	0.76	4.41	0.06	−0.05	0.66	0.30	−0.45
	Sensory Experience Arousal	3.52	0.60	3.30	0.42	3.80	0.75	3.64	0.35	−1.13	−0.93	−1.63	−1.30
	Valence	3.88	1.46	3.58	0.74	3.80	1.25	3.84	0.09	−0.79	0.41	0.30	−0.87
	Emotional Experience Arousal	3.10	0.88	3.22	0.33	3.29	0.45	3.58	0.28	0.75	0.82	−1.13	−1.03
	Number of Strokes	17.87	7.07	17.30	2.12	18.00	0.00	18.15	1.41	−0.27	0.19	−0.10	−0.55
	Number of Meanings	1.22	0.71	1.25	0.71	1.17	0.71	1.30	0.00	0.19	0.76	0.00	−0.53
	Familiarity	5.70	0.86	5.52	0.81	5.54	0.20	5.80	0.05	−0.65	1.04	0.52	−1.21
	Semantic Transparency	6.35	0.12	6.22	0.16	6.28	0.08	6.28	0.01	−0.96	−0.09	0.43	−0.48
	Compositionality	4.99	0.38	4.98	0.37	4.65	1.27	4.88	0.27	0.06	0.98	1.31	0.47
	Number of Synonyms	0.30	0.00	0.95	3.54	0.74	2.83	0.95	2.12	1.11	0.42	−1.09	−0.51
	Number of Antonyms	0.61	0.00	0.65	0.71	0.83	0.00	0.55	2.12	0.13	−0.48	−0.37	0.31

*Note.* ***, *p* < 0.001.

**Table 2:** Descriptive Results of Experiments 1 and 2.

	Primer Type	Character Type	Splittability	Error Rate		RT (ms)	
				*M*	*SD*	*M*	*SD*
Experiment 1	Word	Bound	High	0.07	0.26	651	185
			Low	0.06	0.24	643	188
		Free	High	0.03	0.16	633	192
			Low	0.05	0.23	636	186
	Non-word	Bound	High	0.08	0.28	702	185
			Low	0.08	0.27	702	189
		Free	High	0.04	0.20	673	170
			Low	0.09	0.29	688	178
	“※※”	Bound	High	0.09	0.29	735	181
			Low	0.08	0.28	736	184
		Free	High	0.04	0.19	698	162
			Low	0.09	0.28	717	155
Experiment 2	Word	Bound	High	0.11	0.31	670	196
			Low	0.07	0.25	661	198
		Free	High	0.05	0.22	641	176
			Low	0.08	0.27	650	188
	Non-word	Bound	High	0.08	0.27	669	182
			Low	0.08	0.26	675	177
		Free	High	0.05	0.21	659	160
			Low	0.09	0.29	684	184
	“※※”	Bound	High	0.12	0.32	711	187
			Low	0.11	0.31	715	172
		Free	High	0.06	0.23	688	169
			Low	0.11	0.32	703	171

## Data Availability

The original data and analysis codes are available in the Open Science Framework (OSF) repository (https://osf.io/qfuwa/?view_only=e800c515dd084e539c62fc1b0ee998d8, accessed on 28 June 2025).

## Abbreviations

The following abbreviations are used in this manuscript:

2C-words	two-character words
VOCs	verb–object constructions
LDT	lexical decision task
AoA	age of acquisition
DHH	deaf and hard-of-hearing
SoCs	structures of coordination
SoMs	structures of modification
SoPs	structures of predicates
SSs	supplementary structures
SRCs	syntactic relationships between the constituent characters
RT	reaction time
PrbE	probability of making errors

## Appendix A

**Table A1:** VOC Targets.

Splittability	Character Type	Targets	English Translation
High	Free	做梦/zuo4meng4	to dream (a dream)
		架桥/jia4qiao2	to build a bridge
		盖章/gai4zhang1	to affix a seal
		忍痛/ren3tong4	to bear the pain
		陪酒/pei2jiu3	to host guests with drinks
		搭腔/da1qiang1	to answer back
		戒毒/jie4du2	to quit drugs
		退货/tui4huo4	to return goods
		灭菌/mei4jun1	to sterilize
		建档/jian4dang4	to create a file
		献词/xian4ci2	to write a dedication
		上市/shang4shi4	to go public
		跳槽/tiao4cao2	to job-hop
		会客/hui4ke4	to receive a guest
		付款/fu4kuan3	to make a payment
		加薪/jia1xin1	to get a pay raise
		有余/you3yu2	to have a surplus
		抗震/kang4zhen4	to resist earthquakes
		随俗/sui2su2	to follow local customs
		入席/ru4xi2	to take one’s seat (at a banquet)
		还债/huan2zhai4	to repay a debt
		叫苦/jiao4ku3	to complain of hardship
		注水/zhu4shui3	to add water (to dilute or to fraud)
	Bound	备案/bei4an4	to file a record
		用刑/yong4xing2	to apply torture
		树敌/shu4di2	to make an enemy
		征婚/zheng1hun1	to advertise for a spouse
		参展/can1zhan3	to participate in an exhibition
		发财/fa1cai2	to make a fortune
		整形/zheng3xing2	to have plastic surgery
		赴宴/fu4yan4	to attend a banquet
		降职/jiang4zhi2	to get a demotion
		从业/cong2ye4	to practice a profession
		越境/yue4jing4	to cross the border illegally
		耗资/hao4zi1	to cost (a sum of money)
		碰壁/peng4bi4	to meet with rejection
		失势/shi1shi4	to lose power or influence
		创刊/chuang4kan1	to launch a publication
		接轨/jie1gui3	to align with
		平叛/ping2pan4	to suppress a rebellion
		出炉/chu1lu2	to be released
		服役/fu2yi4	to serve in the military
		脱脂/tuo1zhi1	to degrease
Low	Free	剪彩/jian3cai3	to cut the ribbon (at a ceremony)
		着迷/zhao2mi2	to be fascinated by
		历险/li4xian3	to adventure
		换岗/huan4gang3	to change the guard
		怀恨/huai2hen4	to harbor a grudge
		留级/liu2ji2	to repeat a grade (in school)
		冲浪/chong1lang4	to surf (the waves/the Internet)
		对证/dui4zheng4	to confront (evidence or person)
		游街/you2jie1	to parade a person through streets
		毁容/hui3rong2	to disfigure someone
		插秧/cha1yang1	to transplant rice seedlings
		拉锯/la1ju4	to saw
		供暖/gong1nuan3	to provide heating
		住嘴/zhu4zui3	to hold one’s tongue
		利己/li4ji3	to benefit oneself
		扑鼻/pu1bi2	to assail the nostrils
		摆阔/bai3kuo4	to flaunt one’s wealth
		布防/bu4fang2	to set up defenses
		处世/chu3shi4	to conduct oneself in society
		制冷/zhi4leng3	to refrigerate
		涂鸦/tu2ya1	to graffiti
		候补/hou4bu3	to be a candidate-in-waiting
		悬梁/xuan2liang2	to hang from a beam for studying
	Bound	泄愤/xie4fen4	to vent one’s anger
		成型/cheng2xing2	to take shape
		待机/dai4ji1	to be on standby
		攻坚/gong1jian1	to assault a fortified position
		护航/hu4hang2	to escort
		就诊/jiu4zhen3	to see a doctor
		道贺/dao4he4	to congratulate
		朝圣/chao2sheng4	to go on a pilgrimage
		开庭/kai1ting2	to open a court session
		施威/shi1wei1	to assert one’s authority
		临战/lin2zhan4	to be on the eve of battle
		受贿/shou4hui4	to accept bribes
		求援/qiu2yuan2	to ask for help
		喘息/chuan3xi1	to catch one’s breath
		益智/yi4zhi4	to benefit intelligence
		充饥/chong1ji1	to appease one’s hunger
		戴孝/dai4xiao4	to wear mourning (clothes)
		绝迹/jue2ji4	to become extinct
		破晓/po4xiao3	to dawn (the day breaks)
		质疑/zhi4yi2	to doubt or to challenge
